# Molecular alterations in high‐grade neuroendocrine tumors of the small intestine

**DOI:** 10.1002/path.70004

**Published:** 2025-12-19

**Authors:** Agathe Hercent, Julien Masliah‐Planchon, David Cohen, Remy Nicolle, Jean‐Yves Scoazec, Philippe Ruszniewski, Ivan Bièche, Louis de Mestier, Anne Couvelard, Jerome Cros

**Affiliations:** ^1^ Department of Pathology, FHU MOSAIC, Beaujon‐Bichat Hospitals, AP‐HP Université Paris‐Cité Paris France; ^2^ Department of Genetics Bichat Hospital (APHP), University of Paris Cité Paris France; ^3^ INSERM U1149 Centre de Recherche sur l'Inflammation Paris France; ^4^ Pharmacogenomics Department Institut Curie, PSL Research University Paris France; ^5^ Department of Biopathology Gustave Roussy Cancer Campus Villejuif France; ^6^ Faculté de Médecine, Université Paris Saclay Le Kremlin‐Bicêtre France; ^7^ Department of Gastroenterology and Pancreatology, FHU MOSAIC, Beaujon Hospital, AP‐HP Université Paris‐Cité Clichy France

**Keywords:** ileal neuroendocrine tumors, ileal neuroendocrine carcinoma, high grade tumor, heterogeneity, NET, NEC, molecular profile

## Abstract

High‐grade neuroendocrine tumors of the small intestine are separated into two groups: well‐differentiated neuroendocrine tumors (NETs, high‐grade) and poorly differentiated neuroendocrine carcinomas (NECs). They represent very rare entities, with few molecular data available, and are very challenging to treat. In this study we aimed to describe the molecular profile of these tumors and their spatial and temporal heterogeneity. We collected a national multicenter cohort of high‐grade NETs (14 patients) and NECs (11 patients). DNA and RNA were extracted and somatic point mutations, copy number variations, and gene expression levels were studied using high‐throughput sequencing of a panel of 571 genes and RNA sequencing, respectively. Additional samples to study spatial or temporal heterogeneity were available for 12 patients, leading to a total of 42 samples analyzed. Differential diagnostic markers were confirmed by immunohistochemistry. NECs resemble their counterparts in other organs, with a relatively high tumor mutational burden (TMB) and frequent alteration of *TP53* and *RB1*, together with organ‐specific alterations such as *APC*. In contrast, high‐grade NETs resemble low‐grade NETs, with a low TMB but frequent chromosomic alterations. Transcriptomic analysis confirmed that high‐grade NETs and NECs are two distinct entities, with specific drivers. Serotonin pathway markers were the most efficient to discriminate high‐grade ileal NETs from NECs. Despite variations in the proliferation index, NETs showed little spatial and temporal heterogeneity, suggesting that epigenetic mechanisms play a crucial role in tumor progression. © 2025 The Author(s). *The Journal of Pathology* published by John Wiley & Sons Ltd on behalf of The Pathological Society of Great Britain and Ireland.

## Introduction

Neuroendocrine neoplasms (NENs) have heterogeneous clinical and molecular profiles and can arise from multiple sites throughout the body. They range from well‐differentiated neuroendocrine tumors (NETs) with few genomic events and a good prognosis to poorly differentiated neuroendocrine carcinomas (NECs) with recurrent and distinct molecular abnormalities and a poor prognosis [1, 2]. Digestive NENs are classified according to their histological differentiation (well versus poorly differentiated) and their proliferative index, as assessed by mitotic count or Ki‐67 index (WHO, 2019) [3]. Well‐differentiated NETs are usually low‐grade lesions – Grade 1 (G1: mitotic count < 2/10 high‐power fields [HPFs] and Ki67 index < 2%) or Grade 2 (G2: mitoses 2–10/10 HPF and Ki67 index 3%–20%) and poorly differentiated NECs are always high‐grade lesions – Grade 3 (G3: mitotic count > 20/10 HPF or ki67 index > 20%). However, a recent entity was added to the 2019 WHO digestive classification of neuroendocrine neoplasm: the NETs G3 tumors [3], which display the morphology of NETs, but have a high proliferative index. It seems that these tumors have a better prognosis than NECs, but their response to classical chemotherapy appears lower, and they show a worse outcome than the G2 group. Their molecular characteristics have been studied in the pancreas and lung, and these tumors resemble G1–G2 NETs, most likely developing from low‐grade NETs through the accumulation of NECs‐like molecular defects [2, 4].

In contrast to other sites, small intestine NENs are most often well‐differentiated and of low grade (Si‐NETs G1 or Si‐NETs G2 low). Low‐grade Si‐NETs present frequent deletions, especially of chromosome 18, and less frequently of chromosomes 16q and 9p, as well as gains of chromosomes 4, 5, 14, 16p, and 19 [5, 6, 7]. Point mutations are rare events in Si‐NETs of low grade [8], and the only recurrent mutations are found in *CDKN1B* (8%) [9] and *APC* (7%–23%) [10, 11, 12]. There is almost no molecular data on small intestine high‐grade NENs. In other digestive organs, NECs are characterized by a high tumor mutational burden (TMB) and frequent mutations in *TP53* (90%) and *RB1* (60%). *MYC* amplification and microsatellite instability (MSI) tumors have also been previously reported [2].

In this study we gathered a large national multicenter cohort of small intestine high‐grade NENs, well‐differentiated (*n* = 14) and poorly differentiated (*n* = 11) with the aim to determine their molecular multiomics profile. We used a next‐generation sequencing (NGS) panel of 571 oncology genes to determine single nucleotide variants, copy number alteration, TMB, and MSI status, together with a transcriptomic profiling using RNA sequencing (RNA‐seq). In addition, we studied the spatial and/or temporal intra‐tumor heterogeneity in 11 well‐differentiated tumors.

## Materials and methods

### Ethics approval

This study was performed according to the Declaration of Helsinki [13] and approved by the Institutional Review Board IRB 00006477, the CEERB of HUPNVERSUS, Université Paris Cité, AP‐HP (number 2019‐042).

### Patients and tumors

Patients were enrolled via the French national ENDOCAN‐TENPATH network dedicated to NEN using as inclusion criteria the presence of a high‐grade small intestine NEN (well‐ or poorly‐differentiated, primary tumor, or metastasis) and the absence of a dysplastic or invasive non‐neuroendocrine component. The following clinical characteristics were collected: age, sex, tumor size, pTNM, and primary tumor localization. All histological materials were reviewed by two experienced NEN pathologists (A.C. and J.C.) to confirm NEN diagnosis, evaluate differentiation, and count the Ki‐67 index according to the WHO 2019 guidelines [3]. As Ki‐67 is often extremely low in small intestine NEN, lesions with a Ki‐67 level above 15% were classified as high‐grade tumors and selected for this study. To study intratumor heterogeneity, we also collected, when possible, several tumor blocks for each patient, coming either from the same surgical specimen (different areas) or from previous or subsequent surgeries/biopsies (see Table 2 for details).

When cell proliferation was highly heterogenous, the maximum Ki‐67 level was used to classify the tumor, and this area was sampled for molecular analysis. However, whenever possible, a second area with a low proliferation was also sampled.

Transcriptomic profiles of a previously published control cohort of 21 low‐grade well‐differentiated ileal NETs were also used.

### Immunohistochemistry

Immunohistochemistry was performed using an automated staining system (BOND‐MAX Autostainer, Leica Biosystems, Buffalo Grove, IL, USA) using the following antibodies: chromogranin A (clone DAK‐A3, 1:100 dilution; Agilent, Santa Clara, CA, USA), synaptophysin (clone DAK‐SYNAP, 1:100 dilution; Agilent), CD56 (clone 504, 1:100 dilution; Leica Biosystems), Ki‐67 (clone MIB1, 1:200 dilution; Agilent), p53 (clone DO7, 1:100 dilution; Agilent), Rb1 (clone 4H1, 1:500 dilution; Ozyme, Saint Cyr L’ecole, France), TTF1 (clone 8G7G3/1, 1:500 dilution; Agilent), DDC (polyclonal, Sigma, Darmsadt, Germany), and PAX8 (Clone MRQ‐50, Roche, Boulogne‐Billancourt, France).

### DNA/RNA extraction

Ki‐67‐guided punches were performed in formalin‐fixed paraffin‐embedded (FFPE) tumor block using a 1‐mm needle core. DNA and RNA extraction was performed using the Qiagen Allprep FFPE kit (Chatsworth, CA, USA), following the manufacturer's protocol. DNA and RNA quality was assessed using the Nanodrop quantification system (Thermo Fisher Scientific, Saint Aubin, France). Double‐stranded DNA quantity was assessed using a Qubit® 2.0 Fluorometer (Invitrogen, Eugene, OR, USA).

### Next‐generation sequencing

DNA sequencing was performed using a 571 gene‐panel: the DRAGON‐Dx panel (Detection of Relevant Alterations in Genes involved in Oncogenetics). Libraries were generated with the SureSelect® XT2 kit (Agilent Technologies™) using 50 ng DNA input. Sequencing was performed on a NovaSeq® 6000 (Illumina™, San Diego, CA, USA). Gene mapping was performed using the BWA (v0.7.15, https://bio‐bwa.sourceforge.net/) and Varscan2 (v2.4.3, https://varscan.sourceforge.net/) software. Variant calling was performed with SAMtool (https://www.htslib.org/workflow/fastq). Single‐nucleotide variants and copy number alterations (CNA) smaller than 50 pb were filtered as follows: exonic or splicing variants (+/− 20 pb within the intron) were retained, while synonymous variants were deleted. Only alterations with a frequency lower than 0.1% in the GnomAD database were selected and variant with an occurrence within the same run above 10 were eliminated. Potential mutations were retained when coverage was above 100X and allelic frequency was > 3%. The following *in silico* prediction software were used to predict pathogenicity of the selected variation: SIFT (v.6.2.0, https://sift.bii.a‐star.edu.sg/), Mutation Taster (v.2013, https://www.mutationtaster.org/), PolyPhen‐2 (v.2.2.2r398, http://genetics.bwh.harvard.edu/pph2/), UMD (https://umd-predictor.genomnis.com/), SNP and Go (https://snps.biofold.org/snps-and-go/pages/contact.html), and FATHMM (https://fathmm.biocompute.org.uk/).

Finally, in each sample tumor mutational burden (TMB) was assessed. TMB was defined as the number of nonsynonymous SNV and small in/del in a one megabase (Mb) coding region. As TMB number reported in the literature are typically calculated with data from exome sequencing, we could not use the same threshold. In this panel, a TMB above 15 mut/Mb was considered to indicate a high mutational burden.

### Copy number alterations

The genomic profile was obtained through the next‐generation sequencing data from the Dragon Panel. Chromosomic segments were measured and copy number and/or loss of heterozygosity was inferred through the calculation of the median of each relative copy. CNA were classified as following: 0 allele copy: deep del; 1 copy: del; 2, 3, or 4 copies of the same allele: loss of heterozygosity (LOH); 5 or more copies of the same allele or both allele: high copy gain (HCG). As we were using tumoral tissues, low copy gain (3 or 4 copies were not considered) and 3 or 4 copies of the same allele were annotated LOH, whereas 3 or 4 copies of a region without LOH were considered normal (no CNA).

### RNA expression

The RNA library was prepared from 150 ng RNA, using the Quan'Seq 3’mRNA‐Seq Library Prep Kit FWD (Lexogen, Vienna, Austria) following the manufacturer's protocol, and sequenced on a Novaseq® platform (Illumina, San Diego, CA, USA) to a minimum depth of 10 million reads per sample. Raw FASTQ files were probed for sequencing quality control using FastQC. Sequencing reads were mapped to the human GRCh38 genome using STAR with settings optimized to 3’RNA‐seq data (available on request). Gene expression quantification was performed using FeatureCounts (https://subread.sourceforge.net/featureCounts.html) with default parameters and the Ensembl transcript annotation (Ensembl 101, https://www.ensembl.org/info/genome/genebuild/index.html). Gene counts were normalized using UpperQuartile normalization and log2 transformed.

Principal component analyses (PCA) was used to determine the components with the highest variance using Rstudio (v.1.3.1056, with R version 4.0.2, https://larmarange.github.io/analyse‐R/installation‐de‐R‐et‐RStudio) and R package DESeq2 (https://lashlock.github.io/compbio/R_presentation). PCA analysis was performed on nontransformed RNA‐seq counts, while unsupervised clustering was realized with t‐transformed data, RNA‐seq counts were used. To elucidate subtypes structure, unsupervised clustering was performed on log2‐transformed data using the Euclidean distance metric and ‘complete’ method implemented in the R package heatmap (https://cran.r-project.org/web/packages/ggplot2/index.html). The clustering was based on significant differential expressed genes (DEG) (*FDR* < 0.05) determined using the DESeq2 R package (https://lashlock.github.io/compbio/R_presentation.html), with nonnormalized RNA‐seq counts as input.

Overrepresentation analysis (ORA) was performed using the enrichR package to determine which pathways are enriched by DEG clusters upregulated for different tumor subtypes. Gene sets from Reactome, KEGG, Wikipathways, Gene Ontology Biological Pathway (GO BP), Cellular Component (CC), Molecular Function (MF), and the C6 Oncogenic Signatures from the Molecular Signatures database (MSigDB) collections (https://www.gsea-msigdb.org/gsea/msigdb) were used.

## Results

Twenty‐five patients were selected: 14 with well‐differentiated NETs and 11 with poorly differentiated NECs. All selected tumors were positive for at least two neuroendocrine markers. Among the NECs, 100% were positive for synaptophysin (11/11), ranging from focal expression (two tumors) to strong expression (six tumors). Similarly, all NECs were positive for chromogranin (11/11), with expression ranging from weak (five tumors) to strong (five tumors). Representative H&E images are shown in the supplementary material, Figure S1.

### Genomic landscape of small intestine high‐grade NETs

Details of the samples used for the primary analysis are summarized in Table 1. When multiple samples were available for one patient, we prioritized the naïve primary high‐grade tumor, which was possible for 19 patients. For patients 11 and 20, the primary tumors were obtained after first‐line treatment; therefore, we used the samples from the naïve hepatic metastasis for the primary analysis. Finally, for patients 14, 16, and 21, the primary tumors were low‐grade tumors (ki < 1%). Therefore, the high‐grade hepatic metastases were used instead for the primary analysis.

**Table 1:** Clinicopathological information for the 25 patients including localization of the 25 samples selected for analysis.

Patients	Differentiation	Localization of sample	Ki67 of the selected sample	Primary tumor localization	pTNM
5	PD	Ileal	60	Ileal	pT4N0
6	PD	Ileal	90	Ileal	pT4N1
7	PD	Ileal	80	Ileal	pT4N1
8	PD	Ileal	80	Ileal	pT4N2
9	PD	Ileal	45	Ileal	NA
12	PD	Jejunal	50	Jejunal	pT4
17	PD	Jejunal	90	Jejunal	pT4
18	PD	Ileal	70	Ileal	pT4N0
19	PD	Ileal	70	Ileal	pT4N1
23	PD	Ileal	90	Ileal	pT4
24	PD	Jejunal	60	Jejunal	pT3N1
1	WD	Ileal	15	Ileal	pT4N1
2	WD	Ileal	18	Ileal	pT4N1
3	WD	Ileal	15	Ileal	pT3Nx
4	WD	Ileal	22	Ileal	NA
10	WD	Ileal	25	Ileal	pT3N1
11	WD	HM	15	Jejunal	pT4N1
13	WD	Ileal	20	Ileal	pT4N0
14	WD	HM	35	Ileal	pT4N1
15	WD	Ileal	25	Ileal	NA
16	WD	HM	30	Ileal	pT2
20	WD	HM	18	Ileal	pT4N1
21	WD	HM	20	Ileal	pT3N1
22	WD	Ileal	15	Ileal	NA
25	WD	HM	26	NA	NA

Abbreviations: HM, hepatic metastasis; PD, poorly differentiated; WD, well‐differentiated.

In high‐grade NETs, Ki‐67 ranged from 15% to 25% in primary tumors, tumors mutational burden was low 6.8 (+/− 3.15), and no MSI tumor was detected. In the 11 primary high‐grade NETs, next‐generation sequencing (NGS) did not identify any recurrent pathogenic variant, especially no mutation in *CDKN1B*, suggesting a highly heterogeneous driving mechanism with an important participation of epigenetic drivers (supplementary material, Table S1). Nevertheless, we identified pathogenic mutation in the following genes described in NETs of other localization such as *RICTOR* in the mTOR pathway and *KMT2A* (chromatin remodeling). The other mutated genes did not aggregate in a particular signaling pathway: *IGF2R, FGF3, SDHD, APAF1, CDC27*. One tumor (Ki‐67 15%) showed a stop mutation in *RB1* but without the loss of the other allele.

However, recurrent loss of heterozygosity was identified: Chr 3p25.3 (5/11 including *FANCD2* and *VHL*) Chr 9 (3/11 total and one partial, including *CDKN2B*), the 16p13.3 region including *TCS2* and *PKD1* (6/11), the 17p13.1 region including *TP53* (deletion in 4/11 tumors), Chr 18 (4/11 tumors), the 19p region (4/11), and chromosome 22 (4/11). Two tumors also had a gain of chromosomes 5, 7, and 14 (more than five copies).

### Genomic landscape of small intestine NECs

In NECs, Ki‐67 ranged from 45% to 90% in primary tumors. The mean TMB was higher in NECs (23.6 mut/mb +/− 22.0; 9/11 with a TMB > 10 mut/mb). Two tumors (patients 5 and 8) had an MSI signature without mutations found in MMR genes nor in *POLE* (TMB 77.5 and 427, respectively). Immunohistochemistry of *MSH6/MSH2/PMS2/MLH1* in these two tumors showed no loss of expression.

In primary NECs, NGS identified recurrent mutations in genes that were reported to be altered in NECs from other organs, suggesting more homogeneous carcinogenesis mechanisms. *TP53* was the most commonly mutated gene (5/11 cases) followed by *RB1*, *ERBB4, ROBO1, IG2FR, JAK2, MYO3A, MGA, PKD1, CHD3* in 4/11 cases and *APC*, *mTOR, RICTOR, GLI2, KDR, FAT1, NIPBL, IL6T, NSD1, ARID1B, MLTT4, MET, KMT2A, KMT2C, DNMT1, GATA3, MRE11A, TRAF3, CHD9, EP300, CUL4B* in 3/11 cases. Other genes carried a mutation in only one or two samples. TP53 and RB1 alterations were confirmed by immunohistochemistry (IHC) (supplementary material, Figure S2).

Recurrent deletions were identified encompassing the 16p13.3 region that contains *TCS2* and *PKD1* (8/11), the 17p13.1 region containing *TP53* (7/11), chr18 (5/11) with *SMAD4*, Chr 19 (complete loss in 2/11 and partial loss in 2 tumors containing *STK11*), the Chr 10p region (*GATA3, MYO3A*, 4/11), the chr 5q11.2 region (*IL6T* and *MAP2K1*) (3/11), and chromosome 22 (2/11). One *MYC* amplification was detected in patient 17.

To summarize, the most frequently altered genes in small intestine NECs were the well‐described *TP53* and *RB1* genes, intestinal adenocarcinoma‐like genes such as *APC* and *STK11*, followed by nontumor‐specific tumor promoting genes such as *PKD1*, *TSC2* and *ERBB4*.

### Transcriptomic analysis of small intestine high‐grade NEN

**Figure 1:**
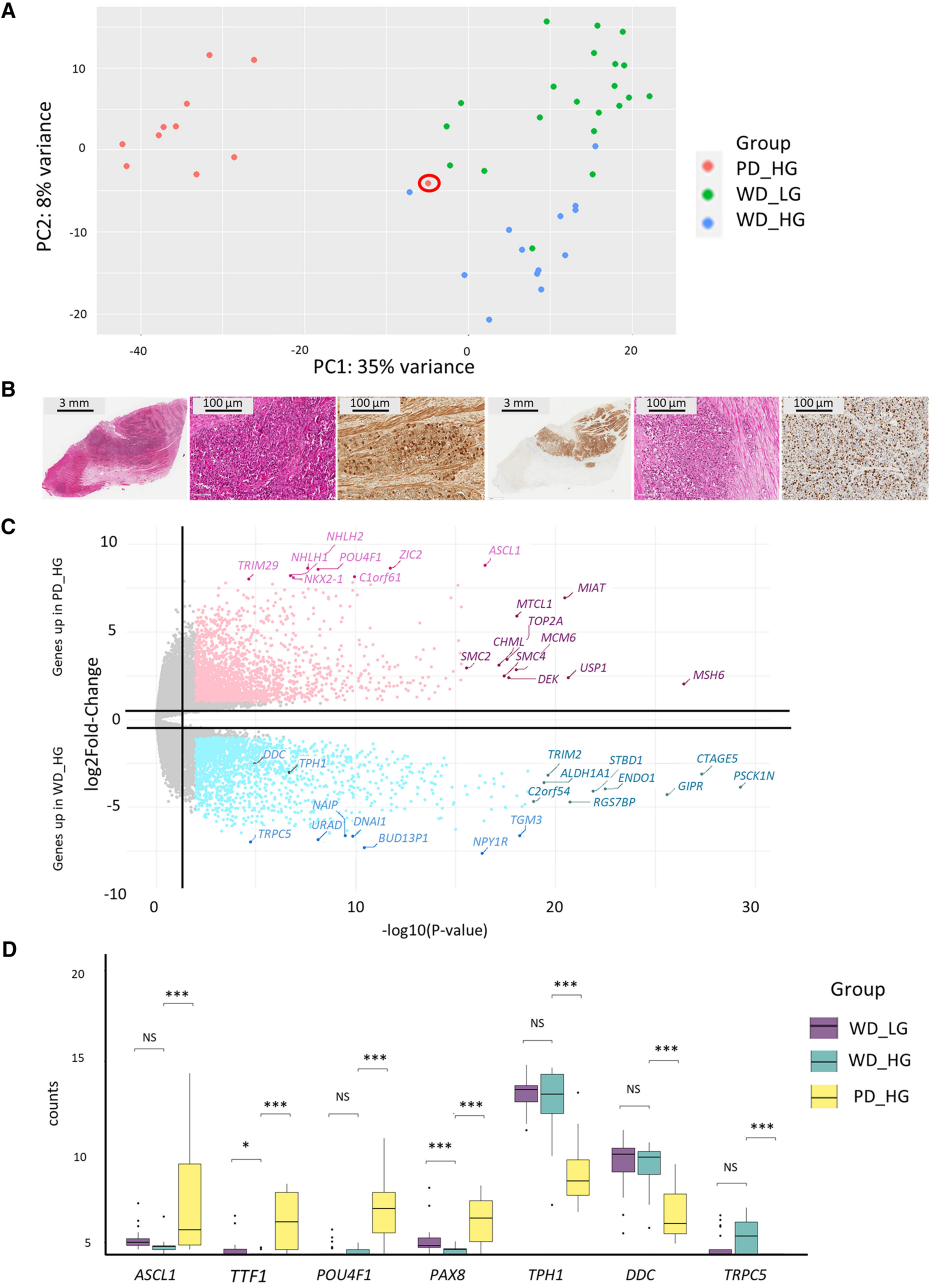
analysis of high‐grade NETs and NECs. (A) Principal component analysis (PCA). The circled sample is the one shown in panel (B). (B) Hematoxylin and eosin (H&E), Ki67 immunohistochemistry (IHC), and serotonin IHC of the outlier neuroendocrine carcinomas (NECs). (C) Volcano plot comparing gene expression in high‐grade neuroendocrine tumor (NETs; lower panel) and NECs (upper panel). (D) Expression plot of NECs (*dASCL1*, *TTF1*, *POU4F1*, *PAX6*) and NETs (*TPH1*, *DDC*, *TRPC5*) markers in well‐differentiated (WD) low‐grade NETs, WD high‐grade NETs, and NECs. NS, not significant; *p < 0.05; **p < 0.005; ***p < 5^e−4^. PD, poorly differentiated; WD, well differentiated; HG, high grade; LG, low grade.

Transcriptomic analysis by RNA‐seq was performed on 13 high‐grade small intestine NETs and 11 NECs. An additional published cohort of low‐grade small intestine NETs was also added for comparison [14]. PCA showed a clear separation of NETs and NECs, even between high‐grade NETs and NECs (Component 1: 35% of the variance) (Figure 1A). The second component tended to separate low‐ and high‐grade NETs (Component 2: 8% of the variance). One NECs sample tended to cluster with high‐grade NETs. Slide reappraisal showed a highly necrotic tumor with an intermediate cell morphology, difficult to classify, showing some areas with an NETs‐like nested architecture, while others were composed of large NECs‐like cell nodules (Figure 1B). In these areas, tumor cells had atypical nuclei with nucleoli suggestive of large‐cell NECs. Ki‐67 was heterogenous, but with large areas counted at 60%. Serotonin expression was conserved, albeit very heterogeneous. This prompted us to evaluate if known markers of NECs in other localizations could be used to distinguish high‐grade NETs from NECs in small intestine together with genes involved in serotonin metabolism or genes highly differential between high‐grade NETs and NECs, such as *TRPC5* (Figure 1C). *ASCL1/NKX2‐1* (i.e. *TTF1*)*/POUF* family genes (*POUF4F1*) reported in lung NECs were indeed highly differential between NECs and high‐grade NETs (Figure 1D). On the contrary, transcripts of key enzymes of the tryptophane pathway, such as *TPH1* and *DDC*, were very low in NECs, suggesting that residual expression of serotonin is in favor of the well‐differentiated nature of the lesion. Finally, immunohistochemistry was performed on proteins involved in highly differentially expressed pathways between NECs and NETs G3: Serotonin and *DDC* (key enzyme of the tryptophan pathway) were highly expressed in NETs G3, with little to no expression in NECs (mean H‐Score 180 versus 0; *p* = 0.01 and 200 versus 0; *p* = 0.001), respectively (supplementary material, Figure S2). In contrast, PAX6 was overexpressed in NECs, albeit at the limit of significance (mean H‐Score 0 versus 160; *p* = 0.07). NKX2‐1 (TTF1) while highly differential at the RNA level showed almost no expression in NECs at the protein level.

**Figure 2:**
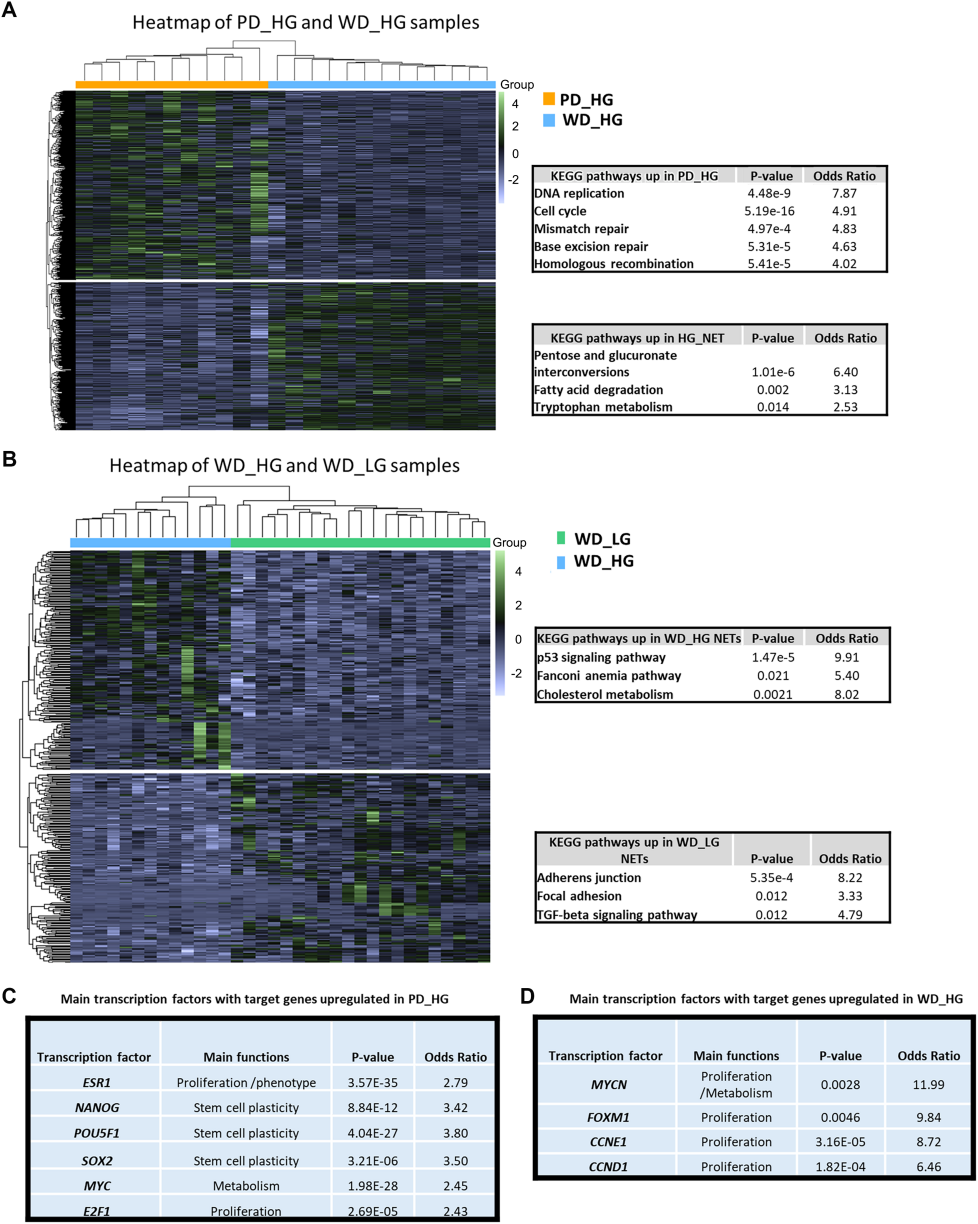
Transcriptomic comparison of small intestine NECs and NETs. (A) Unsupervised hierarchical clustering of high‐grade neuroendocrine tumors (NETs) and neuroendocrine carcinoma (NECs). KEGG pathways upregulated in NECs and high‐grade NETs are shown on the right side. (B) Unsupervised hierarchical clustering of high‐grade NETs and low‐grade NETs. KEGG pathways upregulated in low‐ and high‐grade NETs are shown on the right side. (C) Transcription factors whose target gene set are upregulated in NECs versus high‐grade NETs. (D) Transcription factors whose target gene set are upregulated in high‐ versus low‐grade NETs.

To explore molecular mechanisms driving NECs progression, their transcriptome was compared to those of high‐grade NETs. Enriched pathways in NECs were related to cell cycle, genome stability, and DNA repair (Figure 2A). One of the main transcription factors whose target genes were upregulated in NECs (versus high‐grade NETs) was *ESR1* (Estrogen Receptor 1). This is in line with the strong expression in small intestine NECs of *POU4F1*, another transcription factor that stimulates the binding affinity of the nuclear estrogen receptor ESR1 to DNA estrogen response element (ERE), and hence modulates ESR1‐induced transcriptional activity (Figures 1C and 2B). Other key upregulated transcription factors were *MYC* and the pluripotency stem cell master regulators (OCT4 (POU5F1) / NANOG / SOX2). High‐ and low‐grade NETs showed more related transcriptomic profiles. Proliferation‐related signatures were enriched in high‐grade NETs, while low‐grade NETs were enriched in the neuronal and stromal signature (Figure 2C). The main transcription factors whose target genes were upregulated in high‐grade NETs (versus low‐grade NETs) were involved in proliferation such as *FOXM1* and *MYCN* (Figure 2D).

### Intratumor heterogeneity of small intestine high‐grade NETs

**Figure 3:**
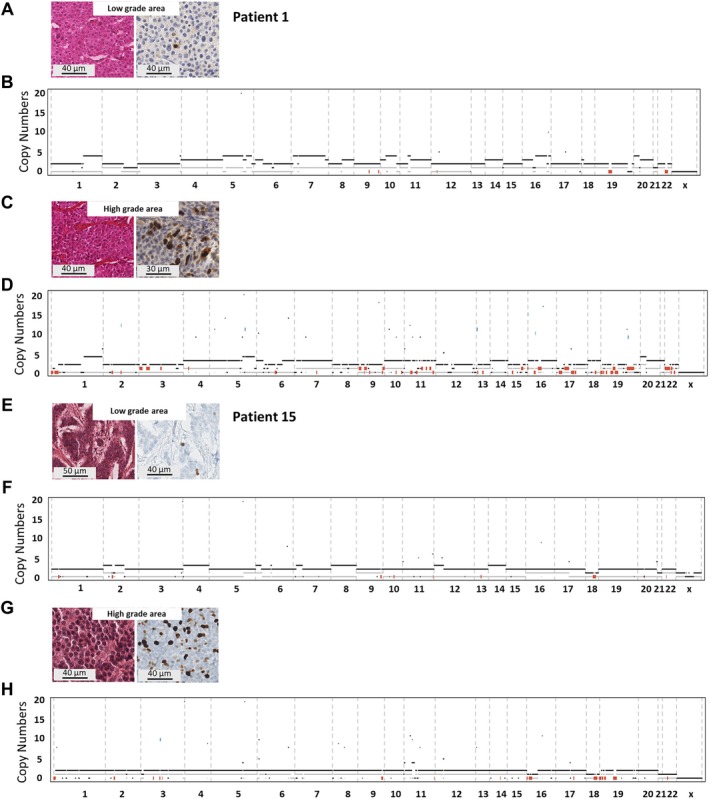
Spatial heterogeneity in tumors from patients 1 and 15. H&E and Ki67 immunohistochemistry (IHC) staining of (A) a low‐grade area and (C) a high‐grade area from the tumor of patient 1. Genomic profile of (B) the low‐grade area and (D) the high‐grade area from the tumor of patient 1. H&E and Ki67 IHC of (E) a low‐grade area and (G) a high‐grade area from the tumor of patient 15. Genomic profile of (F) the low‐grade area and (H) the high‐grade area from the tumor of patient 15.

The Ki‐67 level in high‐grade NETs is often spatially heterogeneous. Among high‐grade NETs, genomic spatial heterogeneity was studied in seven tumors (two samples per lesion high‐ and low‐grade areas) (number of samples per patient and their description is summarized in Table 2). TMB was low and in the same range in the low‐ and high‐grade area of the tumor. In both groups, no recurrent mutations were acquired with the progression to high‐grade. Similarly, no recurrent CNA was more frequent in the high‐grade group and in most cases the same chromosomal losses were present in both samples, suggesting that these genomic alterations arose early in the carcinogenesis process (Figure 3).

**Table 2:** Sample description including patients with one sample analyzed (in gray, *n* = 13) and those with two or more samples analyzed to study spatial heterogeneity (in yellow, *n* = 8) and/or temporal heterogeneity (*n* = 5).

Patient	Differentiation	Sample origin	Heterogenicity	Sample name	Grade	ki 67 (%)
P5	PD	IT	No	PD_ki60_P5IT	HG	60
P6	PD	IT	No	PD_ki90_P6IT	HG	90
P7	PD	IT	No	PD_ki80_P7IT	HG	80
P8	PD	IT (no CNV)	No	PD_ki80_P8IT	HG	80
P9	PD	IT	No	PD_ki45_P9IT	HG	45
P12	PD	JT	No	PD_ki50_P12JT	HG	50
P17	PD	JT	No	PD_ki90_P17JT	HG	90
p18	PD	IT (no CNV)	No	PD_ki70_P18IT	HG	70
P23	PD	IT	No	PD_ki90_P23IT	HG	90
P24	PD	JT	No	PD_ki60_P24JT	HG	60
P19	PD	IT	Spatial	PD_ki35_P19IT	HG area	35
				PD_ki70_P19IT	HG area	70
P4	WD	IT (no CNV)	No	WD_ki22_P4IT	HG	22
P13	WD	IT	No	WD_ki20_P13IT	HG	20
P25	WD	HM (no RNA/No CNV)	No	WD_ki26_P25HM	HG	26
P1	WD	IT	Spatial	WD_ki2_P1IT	HG area	2
				WD_ki15_P1IT	LG area	15
P2	WD	IT	Spatial	WD_ki2_P2IT	LG area	2
				WD_ki18_P2IT	HG area	18
P3	WD	IT	Spatial	WD_ki15_P3IT	HG area	15
				WD_ki0_P3IT	LG area	0
P10	WD	IT	Spatial	WD_ki2_P10IT	LG area	2
				WD_ki25_P10IT	HG area	25
P22	WD	IT	Spatial	WD_ki2_P22IT	LG area	2
				WD_ki15_P22IT	HG area	15
P15	WD	IT	Spatial	WD_ki1_P15IT	LG area	1
				WD_ki25_P15IT	HG area	25
P20	WD	IT after TT	Spatial	WD_ki2_P20IT	LG area	2
				WD_ki35_P20IT	HG area	35
		Initial HM*	temporal	WD_ki18_P20HM	HG	18
P11	WD	Initial HM*	Temporal	WD_ki15_P11HM	HG	15
		JT after TT		WD_ki20_P11IT	HG	20
		HM after TT		WD_ki20_P11HM	HG	20
P14	WD	IT	Temporal	WD_ki2_P14IT	LG	2
		HM*		WD_ki35_P14HM	HG	35
P16	WD	IT	Temporal	WD_ki1_P16IT	LG	1
		LN		WD_ki15_P16LN	HG	15
		HM*		WD_ki30_P16HM	HG	30
P21	WD	IT initial	Temporal	WD_ki1_P21IT	LG	1
		Initial HM* (no DNA/CNV)		WD_ki20_P21HM	HG	20
		HM after 1st TT		WD_ki18_P21HM	HG	18
		HM after 2nd TT		WD_ki40_P21HM	HG	40

*Note*: Samples with a * were the ones selected for the analysis.

Abbreviations: HG, high‐grade; HM: hepatic metastasis; IT: ileal tumor; JT, jejunal tumor; LG, low‐grade; LN, lymph node metastasis; PD, poorly differentiated; TT, targeted therapy (chemotherapy); WD, well‐differentiated.

Temporal/metastatic heterogeneity was studied for five patients (two or three samples per patient, 14 additional samples total) (number of samples per patient and their description is summarized in Table 2). Similar to primary tumor spatial heterogeneity, there was no recurrent mutation nor CNA associated with the metastatic process. Patient 14 presented with a neuroendocrine tumor and synchronous hepatic metastases. Primary tumor was a low‐grade NETs (Ki‐67 = 2%), while the hepatic metastasis was a well‐differentiated but high‐grade lesion (Ki‐67 = 35%) tumor. Both localizations had a low TMB and no pathogenic mutation. The primary tumor showed few genomic alterations with a deletion of the PKD1 and TSC2 region on chromosome 16, a LOH of the 17p region, a deletion of chromosome 18, and a partial deletion of chromosome 19. The metastasis showed a different pattern of CNA with a complete deletion of chromosomes 19, partial deletion of chromosomes 20 and 14, and deep deletion of the following genes: *FANCA, TRAF2, BCL11B, FLT*4 (supplementary material, Figures S3 and S4).

**Figure 4:**
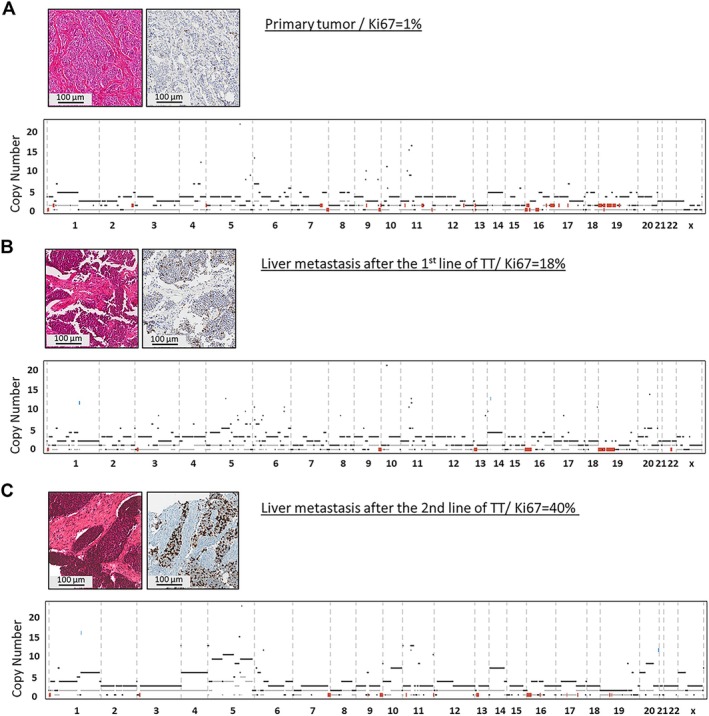
Temporal pre/post‐treatment tumors of patient 21. Hematoxylin and eosin (H&E), Ki67 immunohistochemistry (IHC) and genomic profile of (A) the primary low‐grade pretreatment tumor of patient 21, (B) the high‐grade post 1st treatment liver metastasis, and (C) the high‐grade post 2nd treatment liver metastasis.

Patient 21 presented with a low‐grade primary tumor (Ki‐67 = 2%) and developed hepatic metastasis after his first line of treatment (Ki‐67 = 18%) and another hepatic metastasis after his second line of treatment (Ki‐67 = 40%). TMB was low for all three samples and no pathogenic point mutations were found in the three samples. The three tumors presented a chromosome 8 and 19 deletion and the 16p13.3 deletion, confirming their clonal relationship. The primary tumor and the first relapse shared some common features: Chr13 deletion, chr 10p deletion, and partial chromosome X deletion. These deletions were not present in the second relapse, whose molecular profile was quite different from the other two samples. Interestingly, this more distant sample had the highest Ki‐67 index. However, its genomic profile was still very ‘TNE G3’‐like and did not acquire any recurrent mutation found in the NECs (Figure 4).

## Discussion

In this study we gathered a large multicentric cohort of rare high‐grade NEN of the small intestine. This was possible thanks to the French neuroendocrine pathology network (TENPATH‐ENDOCAN‐PATH) that promote difficult case reappraisal in expert centers or throughout the country, leading to an important database of rare cases. Integrated genomic and transcriptomic analyses confirmed the distinct nature of poorly differentiated carcinomas compared to well‐differentiated NETs, even with a high proliferation index. NECs coming from the small intestine resembled NECs from other organs carrying recurrent mutations in *TP53* (9/11) and *RB1* (6/11) genes and had a higher TMB, as described by Yachida *et al* [15]. In contrast, high‐grade NETs showed few genomic alterations and study of spatial and temporal heterogeneity did not reveal a recurrent genomic driver, suggesting an important role of epigenetic mechanisms in tumor progression.

The analysis of our cohort of high‐grade well‐differentiated NETs showed that they carry very few point mutations in the primary tumors. For this study, samples were sequenced using a large clinical‐grade panel (used in routine diagnostic practice in the Curie Institute), comprising 571 genes of interest in oncology. Additionally, neuroendocrine neoplasms have little stroma and samples displayed a high percentage of tumor cells, limiting the risk of missing genomic events. Furthermore, the threshold used to select variants of interest was low (depth of coverage > 100×; frequency < 0,1% in the gnomAD database; variant allelic frequency > 3%). We therefore believe that there are indeed very few point mutations in these tumors and that their oncogenesis is driven by other events, probably epigenetic. The first exome study of small intestine NETs performed by Banck *et al* [8] showed recurrent mutations in *CDKN1B*, a finding confirmed in other series of low‐grade small intestine NETs (8% and 14%) [8]. No variant was found in *CDKN1B* in our high‐grade NETs. Because *CDKN1B* mutations were reported as driver mutations, this may suggest that well‐differentiated high‐grade NETs oncogenesis follows a different path from low‐grade NETs rather than just additional pro‐oncogenic genomic events on a common path. Our CNA results are inferred from the NGS data, through the calculation of the median of each relative copy. Even though the method has been validated, it is not as precise as a classic CGH array technics and the determination of the exact number of copies may be difficult. However, it is sufficient to detect deep deletion and focal amplification (like in routine practice) and to assess large chromosomal rearrangements to compare samples and follow tumor progression. We have shown that high‐grade NETs also carry recurrent deletions or gains: the chromosome 18 deletion, described by others in NETs of lower grade, present in 35% of our NETsG3 [5]. The mechanism linking oncogenesis and the loss of chromosome 18 in these tumors has been investigated, with the hypothesis that the *SMAD4* and *SMAD2* tumor suppressors played an important role, but the answer is still unclear [16]. Taken together, this may suggest that loss of chromosome 18 is a common early event in small intestine NETs, while *CDKN1B* mutation is a more specific oncogenic path, possibly less likely to progress towards high‐grade NETs, although the size of the cohort is not sufficient to confirm this hypothesis.

The 16p13 deletion, present in 9/11 of our NECs, was also present in 55% of our NETsG3. Interestingly, this deletion has never been described in NEN from the small intestine, whereas it is present in 73% of our NECs and in 55% of our high‐grade NETs. However, similar alterations were reported in pancreatic NETs [17, 18]. This deletion encompasses the *TSC2* gene, a key negative regulator of the mTOR pathway. Missiaglia *et al* [19] demonstrated that TSC2 is downregulated in PanNEN and inversely correlates with prognosis. Indeed, 100% of their PanNECs and 88% of their pancreatic NETs G3 showed a downregulation of *TSC2* in RNA sequencing, confirmed by IHC [19]. TSC2 is also downregulated in PanNETs of lower grade, but less frequently (50%), and the prognosis remains poor compared to PanNETs with intact TSC2. Besides the *TSC2* deletion, we didn't find any common genomic event to explain the proliferation of high‐grade NETs compared to low‐grade NETs. This strongly suggests that epigenetic mechanisms are crucial in small intestine NETs.

Small intestine NEC showed on one hand genomic alterations common to all NEC such as *TP53* and *RB1* and on the other hand mutation of genes specific to the organ such as *APC*, mutated in 28% of our NEC and none of our high‐grade NET. Of therapeutic interest, two of our NEC showed an NGS MSI signature with a high mutational burden but no loss of MMR protein in IHC. This NGS MSI signature was probably detected because of the high mutational burden rather than a true microsatellite instability linked to MMR protein alterations. Nevertheless, these patients might benefit from an immunotherapy rather than conventional chemotherapy, warranting the systematic testing of these tumors in routine practice. These distinct large molecular profiles between high‐grade NETs and NECs is not easily accessible in routine practice. Distinguishing NETs from NECs is of the utmost importance, as it will impact the choice of treatment. Proper assessment of the differentiation may be very difficult, especially on small biopsies or in lesions with a high proliferative index and cellular atypia, especially when combined with large areas of necrosis. This was the case of the tumor presented in Figure 1A, which displayed intermediary histological features and was classified as NECs while its transcriptomic and genomic profile were closer to that of a high‐grade NET. This suggests that biomarkers associated with the differentiation are needed. The assessment of several known NETs and NECs markers in our cohort confirmed that, alone, none is specific enough but combined they may improve tumor classification. For small intestine NEN, expression of serotonin or enzymes of the tryptophan pathway would be in favor of NETs, as shown by the high DDC h‐score in these tumors, while expression of PAX6/TTF1/ASCL1 would be in favor of NECs, as shown by the high PAX6 h‐score demonstrated in IHC. Global transcriptomic analyses confirmed the clear separation between NETs and NECs, a finding similar to pancreatic NEN but different from lung NEN, in which a continuum has been proposed [20, 21]. Proliferation‐related signatures were of course highly differential between NECs and high‐grade NETs and low‐grade NETs, respectively. In addition, key transcription factors involved in stem pluripotency maintenance and self‐renewal (Oct4/NANOG/SOX2) were upregulated in NECs. Together with mutation similar to intestinal adenocarcinomas (i.e. *APC*), this may suggest that some NECs share a common progenitor with carcinoma cells before being reprogrammed toward an aggressive neuroendocrine program, as in lung and prostate [22]. Regarding NETs, the transcriptional differences were less marked but with a strong upregulation of the *MYCN* program in high‐grade NETs, a known oncogene in neural crest‐derived tumors like neuroblastomas, but never reported in NETs [23]. Further confirmation on cellular models will be required.

In this study we also analyzed spatial heterogeneity in NETs G3. In our pairs of samples from primary tumor with high‐ and low‐grade areas, we did not find any event explaining the higher proliferation rate in high‐grade areas. However, the difference of Ki‐67 levels between the low‐grade and the high‐grade area might not have been sufficient to find a strong driving event, or the event might not be detectable with the tools we used. A methylation analysis could bring new clues to explain these hot spots. In addition, the area selection for molecular analyses was focused on tumor cell‐rich areas, preventing the analysis of the microenvironment in tumor progression. Temporal heterogeneity did not show any recurrent driver event either, suggesting that events leading to metastasis or relapse are specific to each patient or the result of epigenetic reprogramming.

## Conclusion

In conclusion, this study gathered a very large series of rare aggressive small intestine NEN, demonstrating that, in this localization, NETs and NECs are clear, distinct biological entities. NECs showed few theragnostic alterations, such as high TMB, warranting a systematic molecular testing of these lesions. In contrast, NETs showed little genomic alteration and heterogeneity, suggesting the importance of epigenetic factors in tumor progression.

## Author contributions statement

JC organized the study and its design. AC, AH and JC collected the samples, analyzed the data, and wrote and reviewed the article. JMP provided the raw data. AC and JC analyzed the DNA raw data. DC and JC analyzed the RNA raw data.

## Supporting information


**Data S1.** Supplementary data
**Figure S1**. Representative H&E of (A) well differentiated NETs G3 (P1, P13, and P15) and (B) poorly differentiated NECs (P5, P18, and P23)
**Figure S2**. Immunohistochemistry of (A) Rb1 (B) TP53, and (C) Serotonin, DDC, PAX6, and TTF1
**Figure S3**. Genomic profile of (A) the primary tumor, and (B) the hepatic metastasis from patient 14
**Figure S4**. Genomic profile of (A) the primary tumor, (B) the post 1st treatment liver metastasis, and (C) the post 2nd treatment liver metastasis of patient 11

**Table S1.** Mutations, tumor mutational burden (TMB), and copy number variations in each sample

## Data Availability

Raw genomic and transcriptomic data are available upon reasonable request to the corresponding author.
