# Supplementary material for: Molecular alterations in high‐grade neuroendocrine tumors of the small intestine
Source: J Pathol. 2025 Dec 19;268(2):215–26. doi: 10.1002/path.70004 (PMC12805610; doi:10.1002/path.70004)
Supplement: Supplementary file 1 — Data S1. Supplementary data Figure S1. Representative H&E of (A) well differentiated NETs G3 (P1, P13, and P15) and (B) poorly differentiated NECs (P5, P18, and P23) Figure S2. Immunohistochemistry of (A) Rb1 (B) TP53, and (C) Serotonin, DDC, PAX6, and TTF1 Figure S3. Genomic profile of (A) the primary tumor, and (B) the hepatic metastasis from patient 14 Figure S4. Genomic profile of (A) the primary tumor, (B) the post 1st treatment liver metastasis, and (C) the post 2nd treatment liver metastasis of patient 11 [file PATH-268-215-s001.docx]

**Molecular alterations in high-grade neuroendocrine tumors of the small intestine**

A Hercent *et al. J Pathol* <https://doi.org/10.1002/path.70004>

**Supplementary Data**

**Supplementary Figures S1–S4**

**Supplementary Table S1 is provided as a separate Excel file**

**Supplementary data**

**Genomic temporal heterogeneity**

Patient 11 presented with a jejunal NETs and a synchronous metastasis at diagnosis. The only pretreatment sample available was a liver metastasis: this sample had a high TMB, with point mutation in the following genes: *DPYD*, *AKT3*, *ASXL2*, *THSDB7*, *TBR1*, *SF3B1*, *ROBO1*, *RASA2*, *ATR*, *PRKCI*, *PIK3CA*, *SLIT2*, *FAT4*, *SNX25*, *PREX2*, *EPK1*, *PTRD*, *TRAF2*, *PTEN*, *ATM*, *NAV3*, *FLT3*, *THBS1*, *MGA*, *FANCI*, *GRIN2A*, *BRD7*, *CHD9*, *MALT1*, *CCNE1*, *EP300*, *ATRX*, *BTK*, *BRCC3*. The tumors also presented numerous CNA with a gain of chromosomes 5, 7, 14, and 17 and a loss of chromosomes 3p25.3 (FANCD2 and VHL), 11q21-11q24.2 (*ATM*, *SDHS*, *KMT2A*, *CHEK1*), chr 13, chr 18, and chr X. The two other available samples were posttreatment: the primary tumors and a novel hepatic metastasis. Interestingly, the novel hepatic metastasis has the same CNA profile as the pretreatment HM, but only one of the point mutation (*EPKK1*). The posttreatment primary tumor showed a similar profile with gain of chromosome 5, 7, 14, and 17 but with less copies (only 3 or 4 copies *versus* 10 to 20 in both HM) and a deletion of chromosome 13, the 11q21–11q24.2 region and chromosome 18. It also shares a two-point mutation with the primary HM (*EPKK1* and *TRAF2*). Two other point mutations were present and private in the sample on *APC* and *ZNF292* (supplementary material, Figure S3).

**
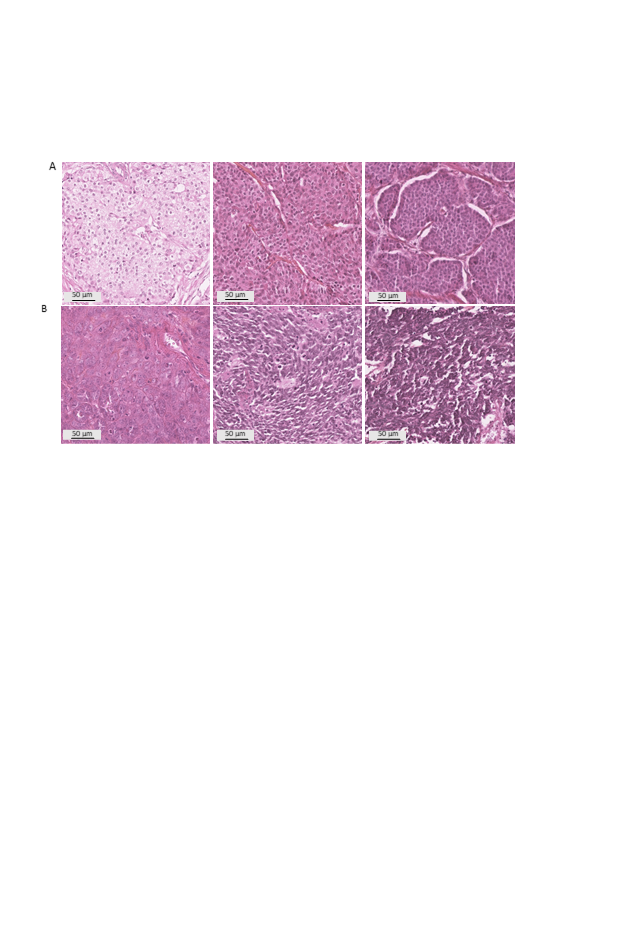
**

**Figure S1. Representative H&E staining of (A) well-differentiated neuroendocrine tumor (NET), grade 3 (G3) (P1, P13, and P15) and (B) poorly differentiated neuroendocrine carcinoma (NEC) (P5, P18, and P23).**

**
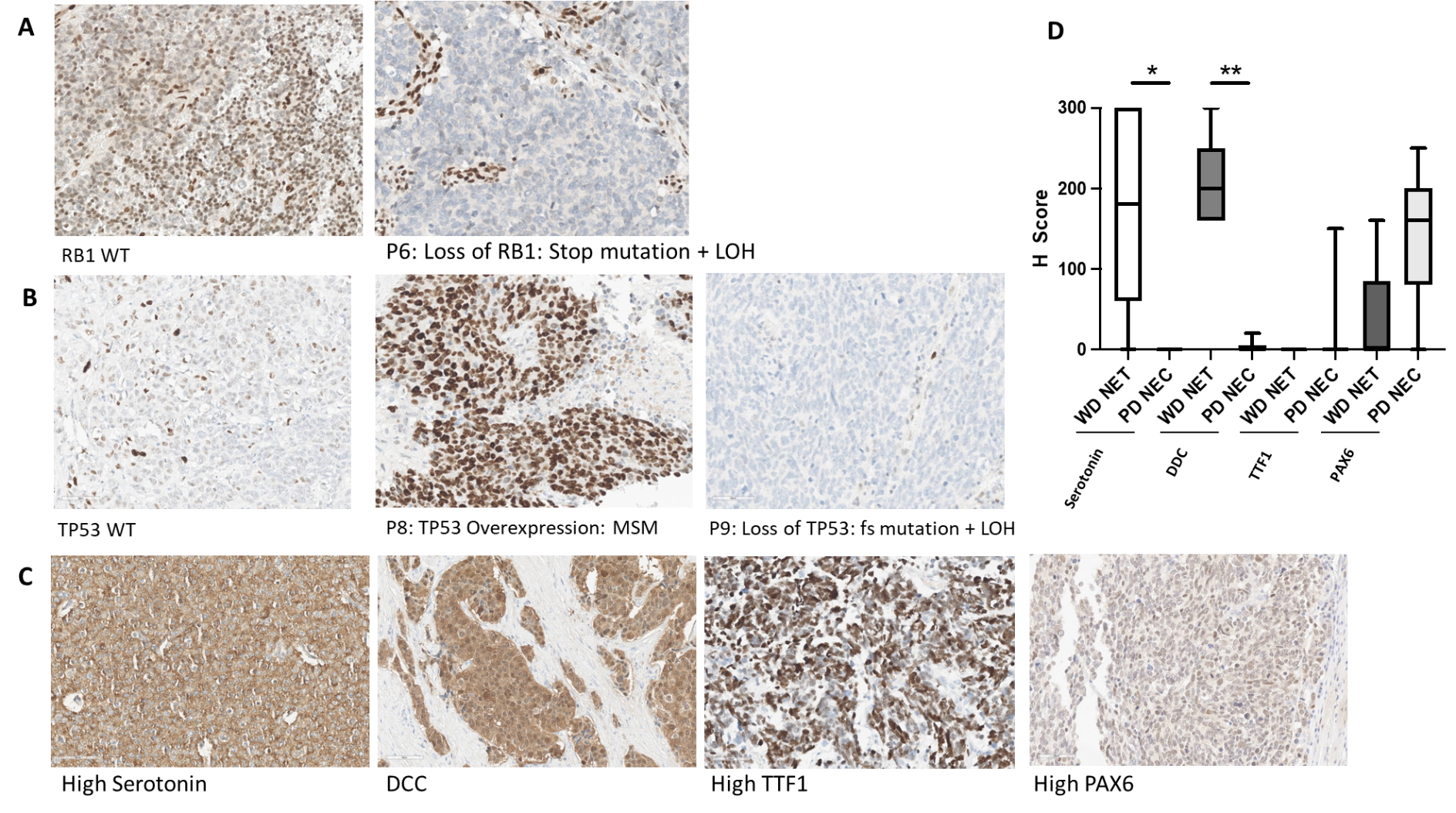
**

**Figure S2. Immunohistochemistry for (A) Rb1, (B) TP53, and (C) serotonin, DDC, PAX8, and TTF1.** (D) Boxplot representing immunohistochemistry results of PAX6, DCC, TTF1 and serotonin between well-differentiated (WD) high-grade neuroendocrine tumors (NETs) and poorly differentiated (PD) neuroendocrine carcinomas (NECs). Fs mutation, frameshift mutation; MSM, missense mutation; LOH, loss of heterozygosity; WT, wildtype.

**
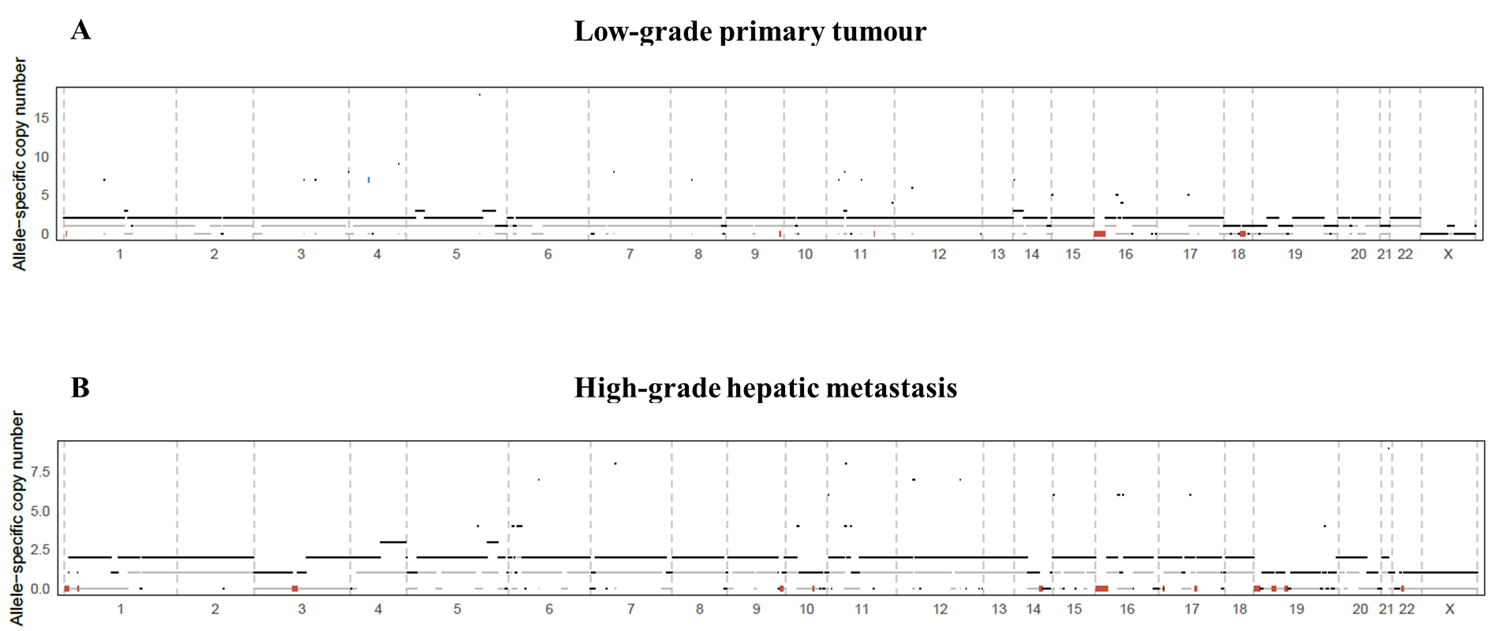
**

**Figure S2. Genomic profile of (A) the primary tumor, and (B) the hepatic metastasis from patient 14.**

**
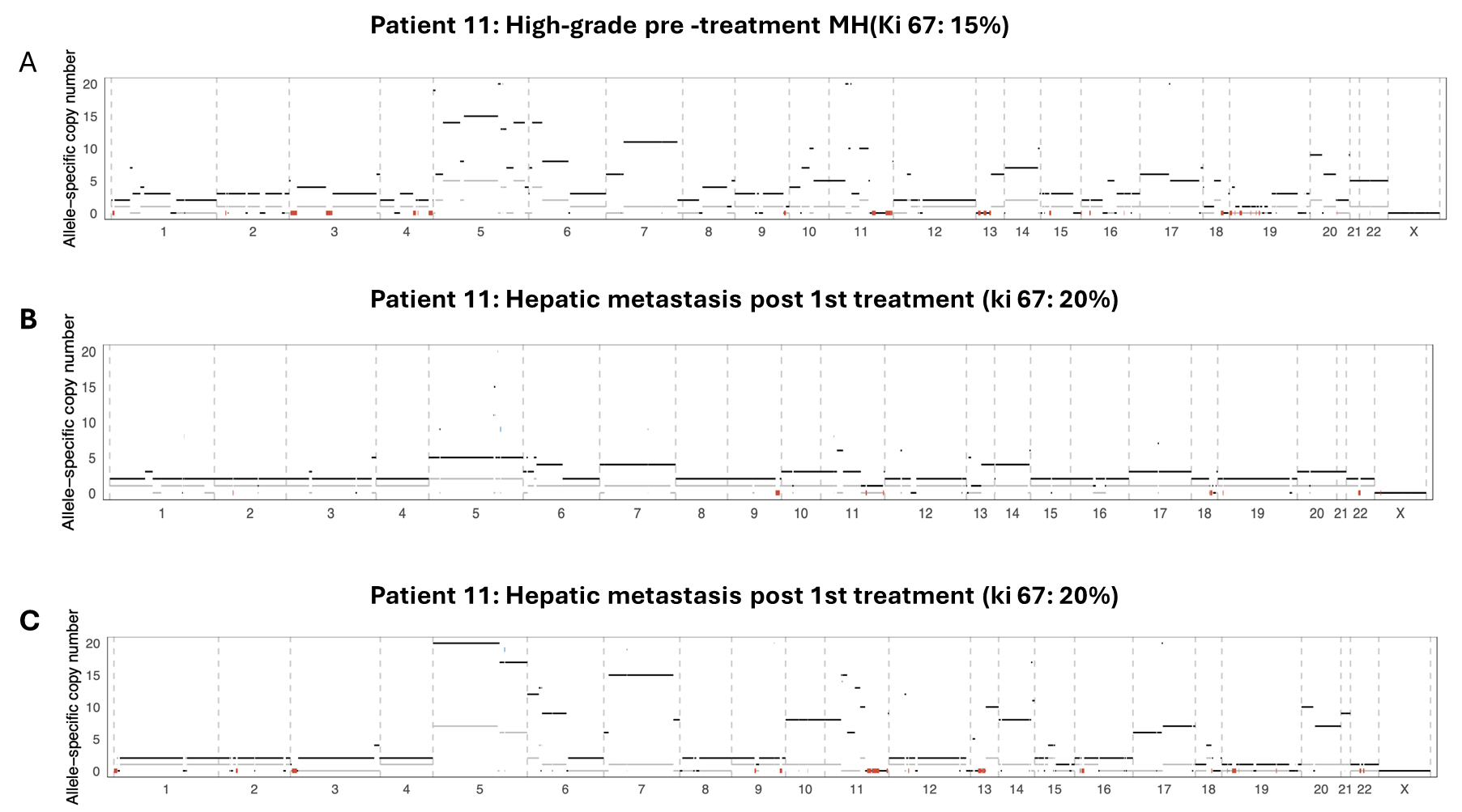
**

**Figure S4. Genomic profile of (A) the primary tumor, (B) the post 1^st^ treatment liver metastasis, and (C) the post 2^nd^ treatment liver metastasis of patient 11.**
